# Oral hygiene and habits of children with autism spectrum disorders and their families

**DOI:** 10.4317/jced.56440

**Published:** 2020-08-01

**Authors:** Simone R. V. Hage, Simone A. Lopes-Herrera, Thais-Helena F. Santos, Danielle A. Defense-Netvral, Aline Martins, Lidiane Y. Sawasaki, Fernanda D. M. Fernandes

**Affiliations:** 1 PhD, Associate Professor. Bauru School of Dentistry, University of Sao Paulo, Bauru, São Paulo, Brazil; 2 PhD. School of Medicine, University of São Paulo, São Paulo, Brazil; 3 Master. Bauru School of Dentistry, University of Sao Paulo, Bauru, São Paulo, Brazil; 4 PhD. Bauru School of Dentistry, University of Sao Paulo, Bauru, São Paulo, Brazil; 5 PhD, Associate Professor. School of Medicine, University of São Paulo, São Paulo, Brazil

## Abstract

**Background:**

Children with Autism Spectrum Disorders (ASD) frequently receive poorer health care then the general population. Frequently the speech-language pathologist is the only health professional that follows the child’s everyday life and therefore is the only resource for guidance regarding basic health habits. Poor oral health may result in severe discomfort and other health problems that can be prevented by simple routine habits and adequate professional follow-up. The aim of the present study was to gather information about oral hygiene and dental care habits of children with ASD and their families. The hypothesis was that these children have poorer oral care habits than their families.

**Material and Methods:**

Participants were parents of 120 children with autism, aged 4 to 12 years in two different cities of the state of Sao Paulo. They answered to a simple questionnaire about oral hygiene and health care habits.

**Results:**

Indicated that there is a significant difference (*p*< 0.001) between the children and their families regarding basic oral hygiene habits, such as brushing and flossing, as well as routine visits to the dentist.

**Conclusions:**

This information clearly indicates the need for education programs aiming to encourage the inclusion children with ASD in the basic habits of oral care carried-out by the families.

** Key words:**Autism disorder, oral hygiene habits, oral health.

## Introduction

Recent research has focused on oral health issues of persons with Autism Spectrum Disorders (ASD). It is clear that oral health-care should be included in all considerations about health care provided to this increasingly large population.

Different approaches are described in the literature and contribute to a comprehensive perspective of the current information about this issue.

In minority countries some studies aimed to describe the oral health situation of children with ASD. Some studies conducted in countries as different as Hong Kong, Sweden, Spain, Netherlands, and United States (1-5) verified that children with ASD had better gingival health and less caries experiences despite poorer hygiene conditions and less visits to the dentist than controls without ASD.

Other studies have focused on procedures destined to facilitate oral health-care procedures with children with ASD. Behavioral problems are usually the biggest challenges and sometimes general anesthesia is the only alternative to guarantee the adequate treatment (6). A behavioral intervention proposal to prepare children with ASD to oral-care was developed in Italy (7) and the authors report that the acceptance rate was associated with the complexity of the care and to the child’s level of verbal fluency. In Hong-Kong (8) researchers observed that the possibility of conducting oral health screening was positively associated with the child’s cognitive, speech and linguistic functioning and negatively associated with challenging behavior.

Studies in majority countries describe challenging realities. In Saudi Arabia, educators, families and health-care providers tend to neglect oral-health care when working with children with ASD (9,10). A research about dental care experiences of children with ASD in three major cities in Saudi Arabia (11) concluded that more than half of the children had no previous dental-care experience and 33% of them were treated under general anesthesia. Studies conducted in India (12,13) concluded that the association between functional limitations and poor oral health is significantly higher in children with ASD; besides that, children with ASD had higher incidence of caries and gingivitis. In Taiwan, early recognition of dental problems in persons with ASD is difficult (14). In Brazil (15), a study reported that children with ASD have more caries, poorer oral hygiene and does not receive adequate oral health-care. According to these studies there is need for more training by the dentists regarding the needs and characteristics of children with ASD.

Parents are the source of information about oral health care and oral health-related behavior in various studies. A recent literature review (16) concluded that family centered approaches based on information provided by parents, their preferences and concerns are the best way to provide oral health care to children with ASD. Another study (17) pointed-out that although parents are the best source of information about how to provide dental health-care to children with autism, there is great individual variability regarding how they accept or react to dental and oral care. These differences had already been identified by a previous study (18) that associated the level of comfort of the parent to their child`s language functioning. Children`s listening abilities were associated to parent`s level of tooth brushing frequency while speech abilities were associated to flossing frequency. A study conducted in India (12) compared the information provided by parents of 270 children with and without ASD and concluded that functional limitations of children with ASD have negative impact on oral health and may affect their quality of life. According to reports by families with a child with ASD and others with normal developing children (10), ASD reduces oral related quality of life of the whole family and apparently leads to neglect of the oral health of unaffected siblings. Parents of children with ASD report less visits to the dentist per year, more rigid choices of toothpaste and specific habits of supervision of tooth-brushing habits (5,19,20).

In Brazil a study (21) with general population identified very high risks (86%) for caries associated to previous caries experience, poor oral hygiene and feeding habits of children. Prior studies (22,23) aimed to verify associations between mother-and-child’s oral and dental health but could not determine any correlation. The relevant factors either for mother’s and children’s dental health were number of daily tooth brushings and flossing habits, feeding habits and dentist appointments. The assessment of social determinants associated to oral and dental health (24) and need for dental treatment indicated that these aspects were similar for mothers and children and were related to educational and income levels.

In Brazil, the Ministry of Health published a document in 2015 about the integral and comprehensive health care available to persons with ASD within the public health system (25). This document, however, do not mention oral health-care to persons with ASD.

The difficulties faced by families with children with ASD in obtaining oral treatment for their children should encourage all other health professionals that work with this population to place some focus on this issue and therefore help preventing dental and gingival problems in this population.

The aim of the present study was to gather information about oral hygiene and dental care habits of children with ASD attending speech-language specialized therapy and their families. The hypothesis is that children with ASD have poorer oral care habits than their families.

## Material and Methods

The institutions’ ethics committees approved the study and its consent form and all participants signed the informed consent form before answering the questionnaire.

Participants were parents of 120 children, aged between 4 and 12 years, diagnosed with ASD and attending speech-language therapy in specialized services in two different cities of the state of São Paulo – Brazil. The sole exclusion criterion was the denial to answer to the questionnaire.

**Figure 1:**
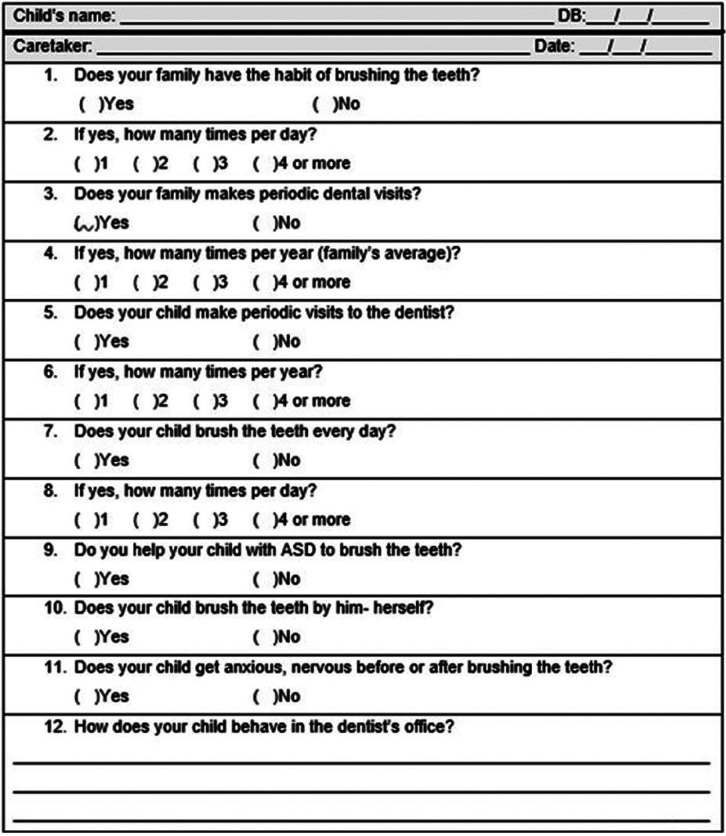
Oral Hygiene and Dental Care Questionnaire.

A brief questionnaire was applied during individual interviews by the child’s speech-language therapist and/or dentist. The questionnaire consisted of 12 questions regarding the families’ and children’s habits of oral hygiene and dental care experiences (Fig. 1).

The statistical analysis aimed to identify associations regarding the children’s age, the hygiene habits, visits to the dentist and the child’s behavior in these situations, using the Chi-square test to compare the answers regarding the children and those regarding the family. All reported results from the Chi-square test presented in this paper had high power of performed test with alpha=0.05.

## Results

The mean age of the children focused in this study was 6 years and 6 months; 20% of them were younger than 5 years and 22% had more than 8 years of age. The questions that demanded yes/no answers received inferential analysis and the results are synthesized in Table 1.

**Table 1:**
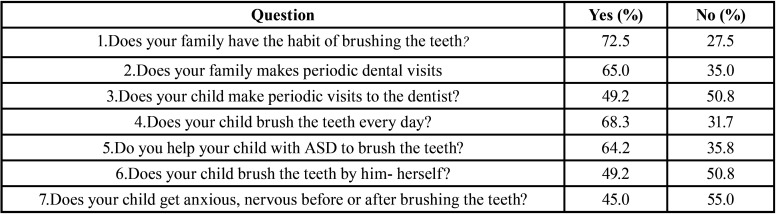
Oral hygiene and dental health habits.

**Figure 2:**
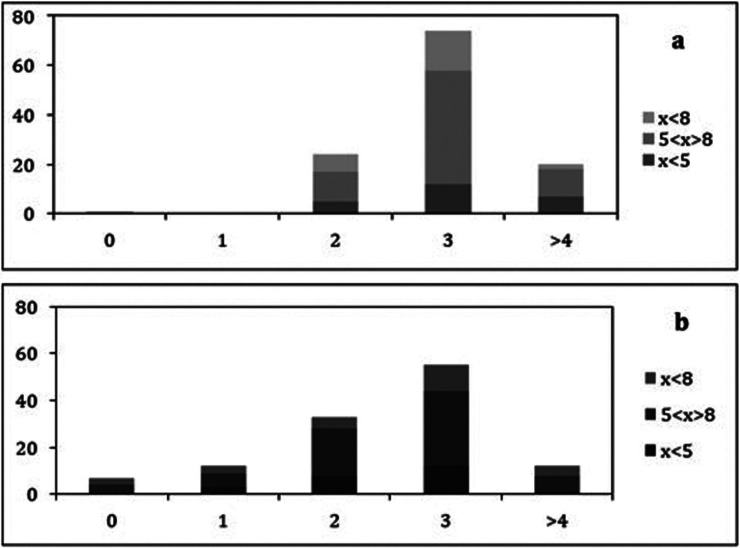
Frequency distribution of the number of times the child with ASD brushes his/her teeth per day (2a) and the number of times their families brush their teeth (2b), for distinct age ranges. Legend: X = age of the children

**Figure 3:**
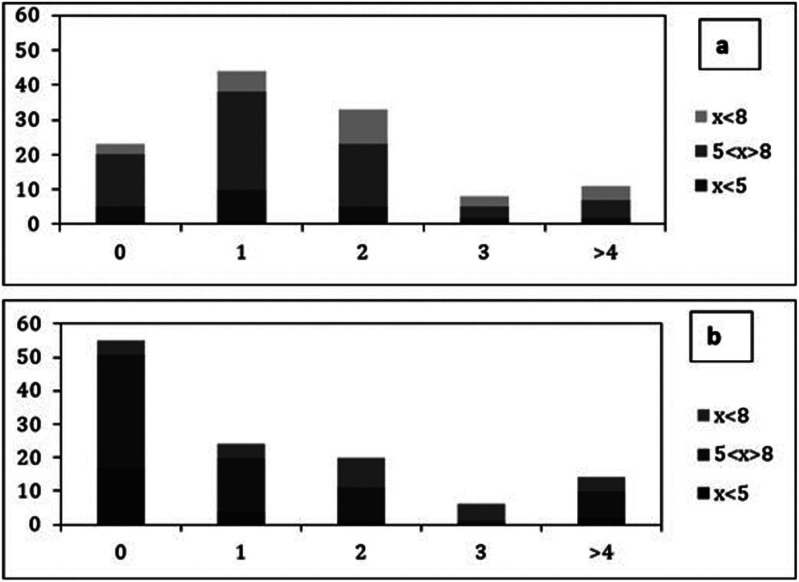
Frequency distribution of the number of times the child with ASD goes to the dentist per year (3a) and the number of times their families go to the dentist per year (3b) for distinct age ranges. Legend: X = age of the children

In what regards the comparison of oral hygiene and health habits, Chi-square analysis indicates that there was significant differences between the number of times the parents report that the child with ASD brushes his/her teeth per day compared to the number of times they do it (Fig. 2), with a calculated chi-square equal to 52.51, with 12 degrees of freedom. There was also a significant difference in the comparison between the number of times they take the child with ASD to the dentist and the number of times the families go to the dentist each year (Fig. 3), with a calculated chi-square equal to 42.02, with 16 degrees of freedom. In both Chi-square tests the *p-value* obtained was less than or equal to 0.001. The parents of 55 children (46.2%) reported that they never took their child to the dentist.

Only 45 parents (37.8%) answered to the question about the behavior of their child during the visits to the dentist. Four children had to be sedated to receive treatment; 6 children showed fear, especially in the first minutes and 13 were restless and agitated. The parents of 22 children reported that they were quiet and collaborative during the dentist appointments and allowed the necessary procedures.

## Discussion

Results indicate that more than 25% of the families do not have the most basic habits of oral hygiene as brushing the teeth or flossing. This information do not agree with a previous study (26) conducted on another large urban area in Brazil, where less than 1% of the adults reported not brushing their teeth at least once a day. Study with Brazilian adults from low socioeconomic status stated that just one man, among the 211 individuals interviewed informed that he doesn’t brush his teeth (27). Such difference in results clearly indicate the need for more data about this specific group, but an international study (10) points out that families with children with ASD tend to neglect health care habits. The numbers regarding children with ASD are even more worrisome since their parents stated that more than 31% do not brush the teeth daily, more than 50% of them do not do it independently but just 64.2% receive help from an adult. These data may be associated to the poorer dental health status of children with ASD in majority countries (11,13).

The differences between data about general population and the families that participated in this study in what refer to visits to the dentist are also relevant. A previous study (27) with adolescents and adults reported that 13.6% of them go to the dentist only when in pain and 15.2% do not go to the dentist at all. In the present research 35% of the families state that they do not make preventive visits to the dentist and it is true to 50.8% of the children with ASD. Dentist appointments were associated to the oral health of mother and children in previous studies (22,23). But the access of children with ASD to dental services is reported to be a challenge in several countries (5,15,20).

The significant difference between the information regarding the families and the children clearly indicate the need for education programs aiming to encourage the adherence to hygienic habits of oral care by families and professionals working with ASD children (7,9).

Contrary to the results of other studies (1,12,16,18), behavioral problems were not described by most parents. Only 11% of the children of the present study were described as being restless and agitated during the visits to the dentist and four have to be sedated to receive dental services. Therefore, the lack of collaboration or behavioral challenges cannot be used to justify the poor oral health care for these children.

The results of this research agrees with a prior study (19) and show that the children with ASD have poorer oral care habits than their families, thus confirming the hypothesis proposed to this study.

Frequently the SLP is the only health professional that follows the child’s everyday life and is faced with the role of providing guidance regarding basic health habits that may improve the child’s general health 

The management of dental/oral health of children with ASD demands careful consideration of the specific needs and available resources (3). Each child should receive the necessary instruction to guarantee the best possible autonomy level regarding oral hygiene and the adequate treatment in timely and efficient manner (17). Understanding of the child’s functioning and difficulties may be useful to improve the dentist’s abilities to address specific needs (18). Practical suggestions, such as written remainders may be useful in implementing habits of oral hygiene (2).

## Conclusions

This study gathered relevant information about oral hygiene and health-care habits of children with ASD and their families. It became clear that the hypothesis that children with ASD receive poorer oral care habits than their families was confirmed. These conditions may be justified by the lack of oral hygiene habits by the adults and also by the fact that preventive visits to the dentist are not part of the health care routine of these families. Some of these issues may be solved by instructional programs that improve the children’s autonomy regarding oral hygiene and by effective oral health policies. The access of children with ASD to services of dentistry is a challenge in several countries and especially in Brazil, where the general population still need systematic information regarding oral care.

The most relevant limitation of this study is the fact that it relies solely on reported information. A more comprehensive study, including clinical examination of oral health status of children with ASD would probably lead to information that could provide elements to public policies.
